# Case Report: Giant axillary lipoma in an infant

**DOI:** 10.3389/fped.2024.1485832

**Published:** 2024-12-09

**Authors:** Haiyang Ren, Haoqiang Lv, Chuanjun Wang, Hao Liu, Xiurui Liu, Hua Wei, Ping Tian

**Affiliations:** ^1^ Department of General Surgery, Linyi Maternity and Child Health Care Hospital, Linyi, Shandong, China; ^2^ Department of General Surgery, Hongxinglong Hospital of Beidahuang Group, Shuangyashan, Heilongjiang, China; ^3^ Department of Gastroenterology, Linyi People’s Hospital, Linyi, Shandong, China

**Keywords:** giant lipoma, axillary, infant, surgery, right

## Abstract

Lipoma is a benign mesenchymal tissue tumor, mainly composed of mature adipose cells; it is most common in adults and is rarely observed in children. The clinical data of an infant diagnosed with a giant axillary lipoma admitted to our hospital were analyzed. A 12-month-old girl presented with a large mass in the right axillary region. Imaging examination suggested a mesenchymal tissue tumor and postoperative pathology confirmed lipoma.

## 1 Introduction

Lipoma, a common benign mesenchymal tissue tumor, is most common in adults and is rarely observed in infants. However, once it occurs, it often has a serious impact on children due to its large size. This report presents a clinical case of a giant axillary lipoma admitted to our department; its diagnosis and treatment processes were retrospectively analyzed, and the literature was reviewed.

## 2 Case description

### 2.1 Patient information

The infant, a 12-month-old girl, admitted our hospital for “5 days after the discovery of axillary tumor.” The parents of the child had inadvertently noticed a tumor under the right axillary, the size of an “egg”; the mass was not associated with pain, redness, fever, or any other discomfort. Concerned about the condition, they came to our hospital for diagnosis and treatment.

### 2.2 Clinical findings

Upon physical examination, a hemispherical mass measuring 6.0 cm × 6.0 cm was observed in the right axilla, with a clear boundary and slightly firm texture. The surface skin appeared normal, without redness or swelling; the mass was active, with an extensive base and no obvious tenderness.

### 2.3 Timeline

The infant had no similar medical history.

### 2.4 Diagnostic assessment

**Figure 1:**
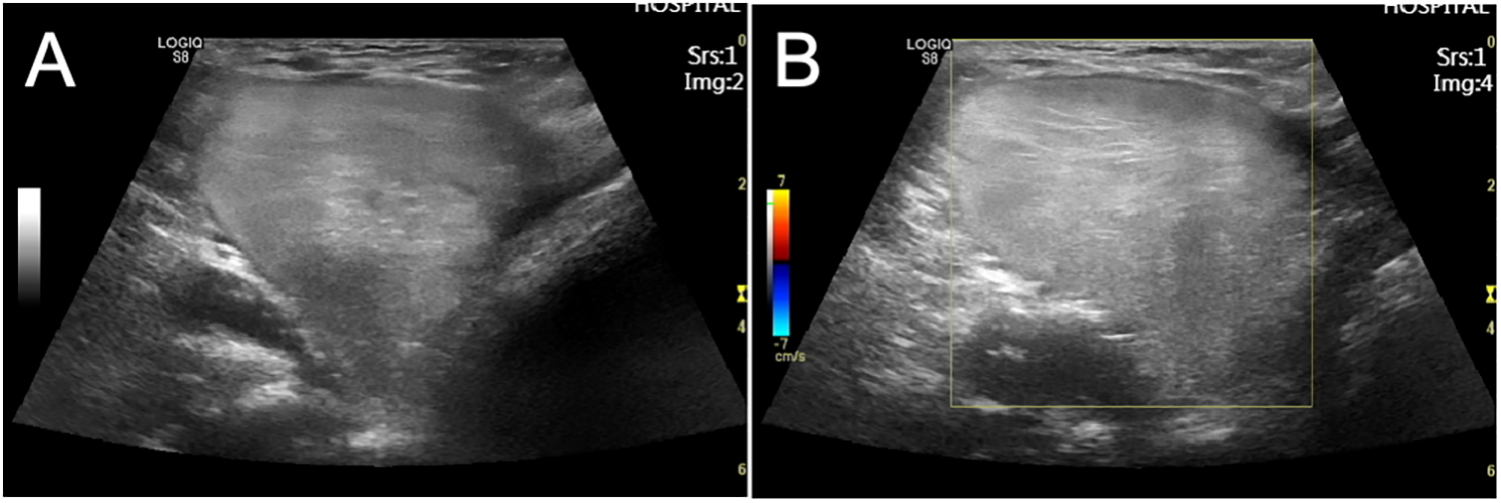
**(A,B)** In the right anterior chest wall near the axillary line, the mass measured 5.1 cm × 4.3 cm × 4.8 cm, with an irregular shape, non-uniform internal echo, and a small amount of blood flow signal on color Doppler ultrasound, suggesting a mesial tissue tumor.

**Figure 2:**
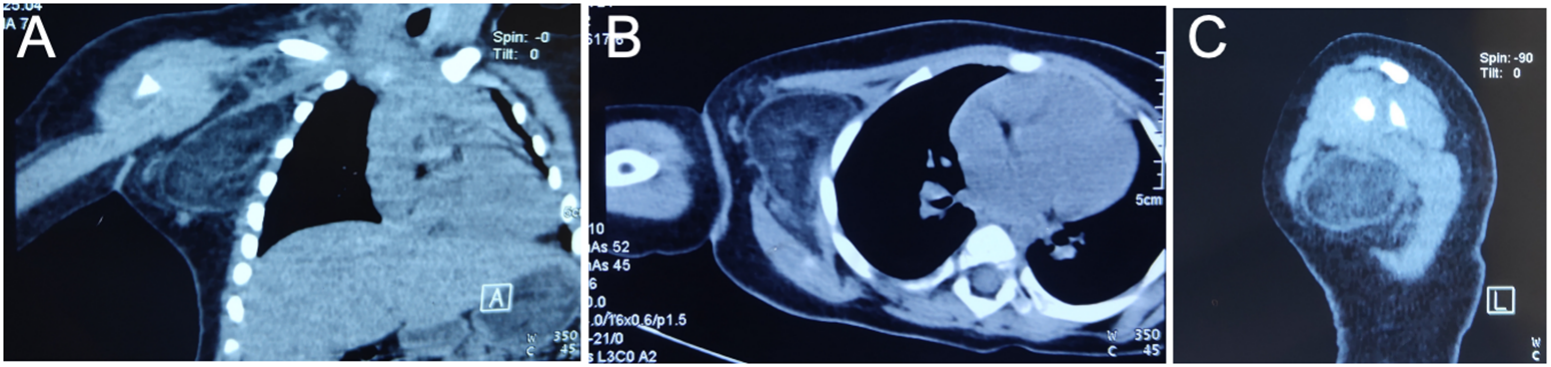
Coronal **(A)**, transverse **(B)**, and sagittal **(C)** plane CT images of the patient's upper extremity prior to excision. A circular low-density lesion was found in the right axilla, characterized by uneven density and clear edges. The CT value was approximately −58 HU, and the cyst wall was smooth.

Laboratory tests included blood routine and C-reactive protein analysis, which gave the following results: white blood cell count of 8.17 × 10^9^/L and a C-reactive protein level of 0.5 mg/L. B-ultrasound examination revealed a mass in the right anterior chest wall near the anterior axillary line, measuring 5.1 cm × 4.3 cm × 4.8 cm; the shape was irregular, the internal echo was not uniform, and the color Doppler ultrasound showed a small amount of blood flow signal, suggesting a mesial tissue tumor (Figures 1A,B). CT examination revealed circular low-density foci in the right axilla with uneven density and clear edges. The CT value was approximately −58 HU, and the sac wall appeared smooth. A sebaceous cyst was considered (Figures 2A–C).

### 2.5 Therapeutic intervention

**Figure 3:**
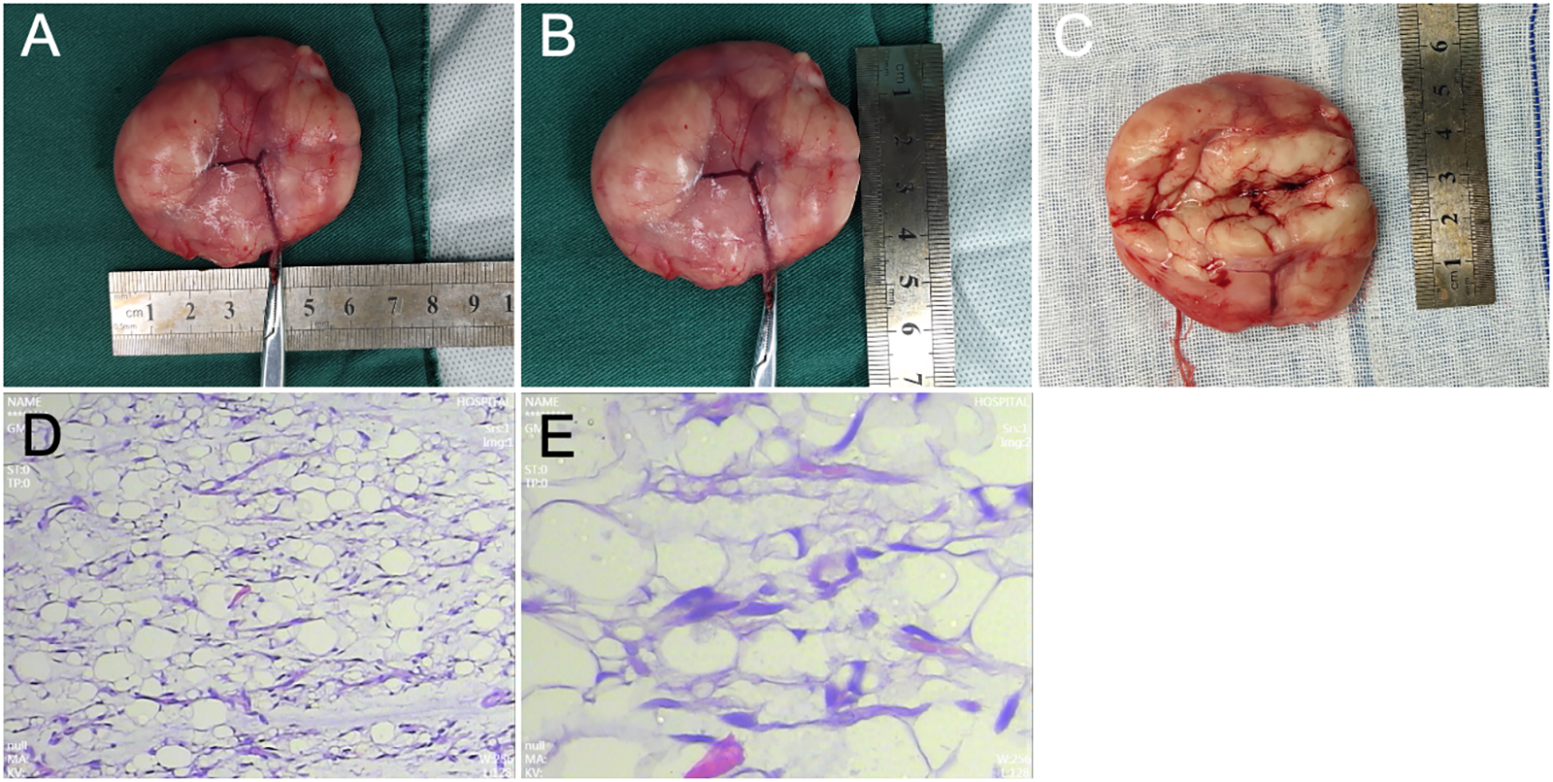
**(A–C)** The mass in the right axilla, with a smooth surface and an envelope, measuring 6.0 cm × 4.5 cm × 2.0 cm. The cross section appears gray, solid, and soft, with a pale and uniform texture and an obvious envelope. **(D)** Under a low-power field-of-view (10×) lens, the tumor is composed of mature fat cells, with slight differences among larger tumor cells; the tumor is divided into irregular lobules by fibrous tissue, with an uneven distribution of capillaries within the tumor tissue. **(E)** High-magnification field (40×) shows mature white fat cells with flattened eccentric nuclei and thin cytoplasm.

Following an improved preoperative examination and the exclusion of contraindications, axillary tumor resection was arranged. After successful general anesthesia, routine disinfection was carried out, the patient was placed in a horizontal position, with the right shoulder elevated, and a 5-cm-long incision was made along the skin grain surface through the skin and subcutaneous tissue. Attention was paid to protecting the subcutaneous blood vessels and nerves; the mass was detected in the subcutaneous tissue, and its edges were blunted. The feeding blood vessels were cut and ligated, and the mass was completely dissected and resected. The mass measured approximately 5.0 cm × 6.0 cm, with a smooth surface, complete envelope, and solid tissue inside. In addition, four lymph nodes were removed simultaneously. The excised tissue was sent for pathological examination. The postoperative pathological findings revealed a right axillary mass with a smooth surface and an envelope, measuring 6.0 cm × 4.5 cm × 2.0 cm. The cross section appeared gray, solid, and soft (Figures 3A–C). Pathological diagnosis confirmed a lipogenic tumor with a tendency to lipoma (Figures 3D,E). Also, four lymph nodes were identified, which were considered to be reactive hyperplasia.

### 2.6 Follow-up and outcomes

The patient recovered well after surgery without significant complications. We followed up with the patient regularly and found that the right axillary incision healed well with no signs of recurrence. The family of the child expressed satisfaction with the surgical outcomes and thanked the medical staff for their attentive care and treatment.

## 3 Discussion

Lipoma is a benign tumor originating in adipose tissue, surrounded by a thin connective tissue cyst containing normal fat cells divided by connective tissue bundles into foliated groups (1). In addition to a large amount of adipose tissue, some lipomas also contain increased amounts of connective tissue or blood vessels, which lead to the formation of complex lipomas. Adipose tissue can give rise to lipomas in any part of the body, although malignant changes are rare. Clinically, lipomas commonly occur under the skin of the head, neck, shoulders, back, and buttocks (2–4). However, they are rare in the axillary area, and even children with axillary lipomas are rarely reported in the literature (5).

Lipomas account for 16% of all benign mesenchymal tissue tumors and are most commonly observed in adults (40–60 years of age), with less than 5% occurring in children (6, 7). Lipomas usually grow slowly, presenting as palpable soft tissue masses; while the majority of lipomas are relatively small, ranging from only a few millimeters to a few centimeters in size, some may grow to larger sizes. Lipomas with a diameter larger than 5 cm are classified as giant lipomas (8–12). Although rare, giant lipomas may appear on the thighs, shoulders, or trunk (13–15). Giant lipomas in the axillary area of infants are very rare. We searched the PubMed database and found no literature reports on giant lipomas in the axillary area of infants. To analyze the epidemiological characteristics of giant lipomas in children, we searched PubMed literature published in English over the last 10 years and reviewed all available reports (16, 17) (Table 1). Eight cases of giant lipomas in children were reported. These included three cases located in the limbs, four in the torso, and one in the head. The clinical characteristics of these giant lipomas are mainly related to their size, and their formation is thought to be triggered by the activation of lipoma tumorigenic factors in the body under the stimulation of various factors, resulting in the abnormal proliferation of normal fat cells. Because lipomas are usually well encapsulated, their treatment is usually straightforward and involves complete surgical removal of the edges.

**Table 1:** Literature review describing the characteristics and treatment of pediatric lipomas.

Reference	Patient age	Sex	Anatomic site	Max. diameter (cm)	Imaging modality	Presenting symptoms	Treatment	Recurrence
Eryılmaz et al. (18)	13 years	F	Cervicothorax	15	MRI	Elevated mass	Resection	None
Vincent et al. (19)	3 months	F	Face	1.5	MRI	Elevated mass	Resection	None
Slavchev and Georgiev (25)	4 years	F	Right forearm	9	MRI	Elevated mass	Resection	None
Gondowardojo et al. (21)	3 years	M	Back	12.7	CT	Elevated mass	Resection	None
Echieh et al. (20)	3 years	M	Chest	18	CT	Elevated mass	Resection	None
Kendrick and Kimble (22)	4 years	F	Gastrocnemius	—	MRI	Difficulty ambulating, intermittent pain	Resection	None
Aihole (23)	6 years	F	Left sternoabdominal wall	20	Ultrasound, CT	Elevated mass	Resection	None
Ai et al. (10)	14 years	F	Left upper extremity	9	MRI	Elevated mass	Resection	None

F, female patient; M, male patient.

“—” indicates information not found in the report.

In this case, the patient was a 12-month-old girl. Her parents inadvertently found a local painless axillary mass in her right armpit, which gradually increased in size and was more than 5 cm in diameter, affecting the life of the child. Concerned about the condition, they sought medical treatment at our hospital, considering that an axillary mass in infants is a problem that needs attention and may be caused by various reasons. These include but are not limited to, lipomas, sebaceous gland cysts, accessory mammary tissue, boils, lymphangiomas, and rarer diseases such as skin B-cell lymphoma. These masses vary in clinical presentation, etiology, and treatment (24) and require a differential diagnosis. Therefore, the identification of an axillary mass in infants requires a comprehensive consideration of multiple factors, including palpatory findings and measurement of the site and depth of the mass, clarity of edges, and mobility, shape, and size of the tumor. Palpation can also reveal the specific location of the tumor. In addition, evaluating the involvement of the nerve trunk and large blood vessels near the tumor area is essential, as it helps judge the deep extent of the tumor. At the same time, benign and malignant tumors should be considered. In general, benign tumors have a diameter of <5 cm, with clear boundaries and the ability to invade deep tissues; in addition, deep benign tumors do not penetrate beyond interfacial zones. In contrast, malignant masses have a diameter of >5 cm, with unclear boundaries and signs of adhesion and tenderness in deep tissues. Finally, relevant medical examinations are required. Therefore, we further included improved laboratory examinations, Doppler color ultrasound, CT imaging, etc., and initially suggested a giant mesial tissue tumor of the armpit; we performed complete resection of the mass, followed by a pathological analysis, which confirmed the diagnosis of lipoma. The successful resection of the giant axillary lipoma in this case not only relieved the pain of the child but also provided us with valuable clinical experience.

## 4 Conclusion

Lipoma, as a common benign mesenchymal tissue tumor, is uncommon in infants and young children. However, once it occurs, it often has a serious impact on children because of its large size. Therefore, early detection, diagnosis, and treatment are crucial in managing such cases. At the same time, we should also emphasize the importance of infant physical examination and timely detection and treatment of potential health problems.

## Data Availability

The original contributions presented in the study are included in the article/Supplementary Material, further inquiries can be directed to the corresponding author.
